# *POLR3A* variants in hereditary spastic paraparesis and ataxia: clinical, genetic, and neuroradiological findings in a cohort of Italian patients

**DOI:** 10.1007/s10072-021-05462-1

**Published:** 2021-07-23

**Authors:** Ilaria Di Donato, Antonio Gallo, Ivana Ricca, Nicola Fini, Gabriella Silvestri, Fiorella Gurrieri, Mario Cirillo, Alfonso Cerase, Gemma Natale, Federica Matrone, Vittorio Riso, Mariarosa Anna Beatrice Melone, Alessandra Tessa, Giovanna De Michele, Antonio Federico, Alessandro Filla, Maria Teresa Dotti, Filippo Maria Santorelli

**Affiliations:** 1 Department of Medicine, Surgery and Neurosciences, University of Siena, viale Bracci, 16, 50055 Siena, Italy; 2 Department of Advanced Medical and Surgical Sciences, University of Campania “Luigi Vanvitelli”, Naples, Italy; 3 Molecular Medicine and Neurogenetics, IRCCS Fondazione Stella Maris, via dei Giacinti 2, Calambrone, 56128 Pisa, Italy; 4 Neurology Unit, Department of Neurosciences, Azienda Ospedaliero Universitaria di Modena, Modena, Italy; 5 Fondazione Policlinico Universitario ‘A. Gemelli’ IRCCS, UOC Neurologia, Rome, Italy; 6 Department of Neurosciences, Università Cattolica del Sacro Cuore, Rome, Italy; 7 Medical Genetics, Università Campus Bio-Medico, 00128 Rome, Italy; 8 Sbarro Institute for Cancer Research and Molecular Medicine, Center for Biotechnology, Temple University, Philadelphia, PA USA; 9 Department of Neurosciences, Reproductive and Odontostomatological Sciences, Federico II University, Naples, Italy

**Keywords:** *POLR3A*, Cerebellar ataxia, Hereditary spastic paraplegia, Leukodystrophy, Spastic ataxia

## Abstract

Mutations in *POLR3A* are characterized by high phenotypic heterogeneity, with manifestations ranging from severe childhood-onset hypomyelinating leukodystrophic syndromes to milder and later-onset gait disorders with central hypomyelination, with or without additional non-neurological signs. Recently, a milder phenotype consisting of late-onset spastic ataxia without hypomyelinating leukodystrophy has been suggested to be specific to the intronic c.1909 + 22G > A mutation in *POLR3A*. Here, we present 10 patients from 8 unrelated families with *POLR3A*-related late-onset spastic ataxia, all harboring the c.1909 + 22G > A variant. Most of them showed an ataxic-spastic picture, two a “pure” cerebellar phenotype, and one a “pure” spastic presentation. The non-neurological findings typically associated with *POLR3A* mutations were absent in all the patients. The main findings on brain MRI were bilateral hyperintensity along the superior cerebellar peduncles on FLAIR sequences, observed in most of the patients, and cerebellar and/or spinal cord atrophy, found in half of the patients. Only one patient exhibited central hypomyelination. The *POLR3A* mutations present in this cohort were the c.1909 + 22G > A splice site variant found in compound heterozygosity with six additional variants (three missense, two nonsense, one splice) and, in one patient, with a novel large deletion involving exons 14–18. Interestingly, this patient had the most “complex” presentation among those observed in our cohort; it included some neurological and non-neurological features, such as seizures, neurosensory deafness, and lipomas, that have not previously been reported in association with late-onset *POLR3A*-related disorders, and therefore further expand the phenotype.

**Supplementary Information:**

The online version contains supplementary material available at 10.1007/s10072-021-05462-1.

## Introduction

*POLR3*-related disorders are a group of clinically overlapping disease entities caused by recessive mutations in the *POLR3A*, *POLR3B*, *POLR1C*, and *POLR3K* genes, which encode subunits of human RNA polymerase III (Pol III), an enzyme involved in the synthesis and translation of several forms of RNA. The best recognized phenotypes consist of severe early-onset hypomyelinating leukodystrophy manifesting with variable combinations of cerebellar ataxia, tremor, spasticity, dystonia, neurodevelopmental regression, oligodontia, and hypogonadotropic hypogonadism [1]. *POLR3A* encodes a catalytic subunit of Pol III. Mutations in this gene are associated with the greatest phenotypic heterogeneity. Other than severe childhood-onset hypomyelinating leukodystrophy, variants in *POLR3A* have been associated with milder, late-onset gait disorders with central hypomyelination, and with parkinsonism dystonia with basal ganglia involvement, with or without non-neurological signs [2–4]. *POLR3A* mutations have also been reported in rare cases of autosomal recessive neonatal progeroid syndrome [5] and in a single case of severe infantile generalized dystonia and hypotonia, leukocytosis, and metabolic acidosis with a lactate peak on brain magnetic resonance spectroscopy [6]. Recently, a milder phenotype consisting of late-onset spastic ataxia has been suggested to be specific to an intronic mutation (c.1909 + 22G > A) in the *POLR3A* gene [7–13]. Interestingly, brain and spine MRI in most patients showed superior cerebellar peduncle (SCP) hyperintensity and spinal cord atrophy without white matter lesions.

Here, we present a series of 10 patients from 8 unrelated families with *POLR3A*-related late-onset spastic ataxia, all presenting with the c.1909 + 22G > A variant in compound heterozygosity, and discuss genotype–phenotype correlations in this cohort.

## Methods

### Patients

In a multicenter study aimed at uncovering the genetic etiology of undefined patients with inherited ataxia and associated disorders [14], we collected 10 consecutive patients from 8 unrelated Italian pedigrees recruited at different third-level neurological centers. The individuals had a previous diagnosis of either cerebellar ataxia or hereditary spastic paraplegia (HSP) of unknown origin. They underwent clinical evaluations at the neurological services in our institutions as part of the routine diagnostic pathway for cases with suspected inherited forms of spastic ataxia. The patients were assessed using a standardized evaluation form. The following categorical scale of disability was applied: 0 = no functional handicap; 1 = no functional handicap but neurological signs on examination; 2 = mild, able to run, unlimited walking; 3 = moderate, unable to run, limited walking without aid(s); 4 = severe, walking with 1 stick; 5 = walking with 2 sticks; 6 = unable to walk, requiring a wheelchair; and 7 = confined to bed [15]. A potential disease progression index was calculated as the ratio between the level of disability (from 0 to 7) and the disease duration in years. MRI studies were performed for diagnostic purposes at multiple centers using 1.5-T MRI scanners and applying standard clinical protocols. All scans were evaluated by the same experienced neuroradiologist.

All the patients and their parents signed an informed consent document and the study was approved by the local ethics committees.

### Genetic analyses

In all the patients, all other potential causes of ataxia and spasticity (i.e., toxic, inflammatory, and metabolic) were ruled out prior to embarking on genetic investigations. Genetic analysis for different spinocerebellar ataxias (SCA1, 2, 3, 6, 7, 17), DRPLA, Friedreich ataxia, and *FMR1*/FXTAS were negative prior to this study. Clinical whole-exome sequencing (in two patients, cases 4 and 10) and next-generation sequencing (NGS) with a targeted multi-gene panel (in the remaining eight patients) were performed using methodologies and a sensitive bioinformatics pipeline that we have already described elsewhere [16]. Segregation studies were performed in nine living parents with written informed consent. Missense variants were classified as pathogenic or likely pathogenic according to their predicted deleteriousness, as established using multiple bioinformatics tools, and prioritized on the basis of a Combined Annotation Dependent Depletion (CADD) score > 25 [17] and the established recommendations of the American College of Medical Genetics and Genomics (ACMG) [18]. Sanger sequencing in each index case and in the parents (when available) confirmed all variants. To corroborate the presence of an intragenic gene rearrangement in patient 4, we developed a quantitative real-time (qRT)-PCR method for use in DNA from the index case and available family members.

## Results

The following features and findings were observed in 10 patients, from eight unrelated kindred, with bi-allelic pathogenic variants in *POLR3A*.

### Clinical features

The 10 index cases (five men and five women) in this cohort had heterogeneous clinical, imaging, and genetic characteristics (summarized in Table 1).

**Table 1:** Clinical and genetic findings in our cohort of patients

	Patient 1	Patient 2	Patient 3	Patient 4	Patient 5	Patient 6	Patient 7	Patient 8	Patient 9	Patient 10
Gender	M	F	M	M	F	F	M	F	M	F
Weight (kg)	Unknown	64	65	80	55	78	75	52	58	62
Height (cm)	Unknown	170	160	169	168	155	175	170	174	168
First variant	c.1909 + 22G > A	c.1909 + 22G > A	c.1909 + 22G > A	c.1909 + 22G > A	c.1909 + 22G > A	c.1909 + 22G > A	c.1909 + 22G > A	c.1909 + 22G > A	c.1909 + 22G > A	c.1909 + 22G > A
Second variant	c.1031G > T (p.Arg344Leu)	c.2788-2A > T	c.2394 T > A (p.Cys798*)	Deletion of exons 14–18	c.3201_3202delGC (p.Arg1069fs*2)	c.4073G > A (p.Gly1358Glu)	c. 4073G > A (p.Gly1358Glu)	c.3733C > T (p.Arg1245*)	c.3733C > T (p.Arg1245*)	c.2554A > G (p.Met852Val)
CADD score	32	NA	39	NA	NA	28.8	31	42	42	37.8
Family inheritance	AR	sporadic	sporadic	AR	Unknown (adopted)	AR	AR	AR	AR	sporadic
Age at onset (years)	16	49	30	13	29	13	15	36	22	19
Age at examination (years)	30	59	54	35	38	64	58	47	41	32
Symptom(s) at onset	Gait imbalance	Gait imbalance	Gait imbalance	Gait imbalance and seizures	Gait imbalance	Gait imbalance	Gait imbalance	Head titubation	Gait imbalance	Gait imbalance
Scale of disability, grade	3	3	3	3	2	5	5	3	3	3
Diesease progression index	0.2	0.3	0.12	0.14	0.22	0.1	0.12	0.27	0.16	0.25
Pyramidal signs										
UL/LL spasticity	-	+	+	-	-	+	+	+	+	+
UL/LL tendon reflex	-	+	+	-	-	-	-	+	+	+
Extensor plantar reflex	-	+	+	+	+	+	+	+	-	+
Cerebellar signs										
Dysarthria	+	-	-	+	+	+	+	+	+	-
Intentional tremor	+	-	-	+	-	+	+	+	-	+
Gait ataxia	+	-	+	+	-	+	+	-	-	+
Cognitive deficits	-	-	-	-	-	-	-	-	-	-
Neurophysiology										
Nerve conduction study	-	-	-	-	-	-	-	-	NA	-
Electromyography	-	-	-	-	-	-	-	NA	NA	-
Motor evoked potentials	NA	+	+	+	+	+	+	+	-	NA
Somatosensory evoked potential	NA	+	+	+	+	+	+	+	+	NA
Dental abnormalities	-	-	-	-	Lack of third molars	-	-	-	-	-
Myopia	+	-	-	-	-	-	-	-	-	-
Acrocyanosis	-	-	-	-	-	-	-	Chilblains	Chilblains	-
Other findings	Atrial sept defect (surgically treated at birth)	Plummer thyroid adenoma, uterine leiomyomas	Popliteal cysts, scoliosis, knee arthrosis, gastritis	Subclinical hypogonadism (low FSH/LH), seizures, lipomas, high- frequency neurosensory deafness	-	-	-	Hammer toes	-	-
Brain and spine MRI										
Spinal cord atrophy	-	NA	NA	NA	-	NA	-	+	+	+
Cerebellar atrophy	+	+	-	-	+	-	-	-	+	+
Thin corpus callosum	-	+	-	-	-	-	-	+	+	-
Superior cerebellar peduncle hyperintensity	-	+	+	+	-	+	+	+	+	-
Corticospinal tract hyperintensity	-	+	+	+	-	-	-	-	-	-
Hypomyelination	-	-	-	-	-	+	-	-	-	-

*CADD* Combined Annotation Dependent Depletion, *AR* autosomal recessive, *UL/LL* upper limb/lower limb, *FSH/LH* follicle-stimulating hormone/luteinizing hormone, *NA* not assessed, *M* male, *F* female, *UL* upper limbs, *LL* lower limbs

Their median age at examination was 39,5 + / − 12,3 years, and their age at the last follow-up ranged from 38 to 64 years. Age at onset of the first symptoms ranged from 13 to 49 years (median age 14,5 + / − 2,1). Gait imbalance was the presenting symptom in most of the cases (9/10). Most were able to walk independently at the time of examination (grade 2 or 3 on the scale of disability), while two required a walker (grade 5 disability). Patients 1 and 4 presented an almost “pure” cerebellar phenotype, whereas patient 2 had a pure HSP phenotype; the remaining cases presented mixed ataxic/spastic features. Dysarthria was present in 7/10 patients; six of the 10 patients showed intentional tremor and in two subjects we noticed head tremor. *POLR3A*-related non-neurological findings typically described by others [10] (i.e., acrocyanosis and dental abnormalities) were absent in all these Italian patients, with the exception of patient 4 who presented subclinical hypogonadotropic hypogonadism with low levels of FSH and LH. No patient showed clinically overt signs of gonadal dysfunction or cognitive abnormalities. Nerve conduction studies and electromyography were unremarkable in all the patients who underwent neurophysiological examinations, whereas motor and somatosensory evoked potentials, when examined, were pathological. One patient (patient 4) presented drug-responsive generalized epilepsy, high-frequency neurosensorial deafness, and lipomas.

### Brain and spinal cord MRI features

**Fig. 1:**
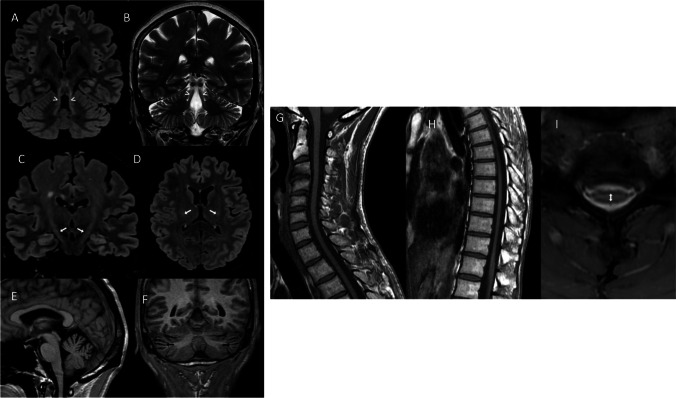
Brain and spinal cord MRI findings in two patients. *Patient 2 A–D*: Hyperintense signal of superior cerebellar peduncles (arrowheads) on coronal 3D T2-FLAIR (**A**) and T2-weighted (**B**) sequences; hyperintensity of corticospinal tract (white arrows) on coronal (**C**) and axial (**D**) 3D T2-FLAIR sequences. *Patient 9 E–I*: thin corpus callosum (**E**) on sagittal T1-weighted sequence; cerebellar atrophy (black arrows) on coronal 3D T1-weighted sequence (**F**). Cervical (**G**) and thoracic (**H**) spinal cord atrophy on sagittal T1-weighted and axial MERGE T2-weighted sequences (the latter measured at the level of cervical enlargement—arrow: transverse diameter of 4.3 mm) (**I**)

The main findings on brain MRI were bilateral SCP hyperintensity on FLAIR sequences (observed in 7/10 patients), cerebellar atrophy (5/10), and spinal cord atrophy (3/6). Other MRI findings included hypoplasia of the corpus callosum (3/10) and corticospinal tract hyperintensity (3/10). A single case (patient 6) exhibited central hypomyelination. Figure 1 shows key MRI features in two of the patients.

### Genetic findings

In our cohort, the so-called common c.1909 + 22G > A splice site variant in *POLR3A* occurred in all the patients, invariably in compound heterozygosity with one of six additional variants (three missense, two nonsense, one splice site), or, in the other case, with a novel large deletion leading to a frameshift and premature protein truncation) (Table 1). The different single-nucleotide variants found in *POL3RA* in our study cohort were classified as likely pathogenic according to the ACMG guidelines.

At first examination, patient 4 appeared to carry the homozygous “common” mutation. However, on the basis of absence of precise segregation in the family, and also through closer analysis of NGS traces and read depth, we were able to detect a possible intragenic rearrangement encompassing exons 14–18 of *POLR3A*, which was confirmed by a qRT-PCR analysis (see Supplementary Figure) in both the patient and his father, but not in his mother, who did carry the “common” variant.

## Discussion and conclusion

Bi-allelic pathological variants in *POLR3A* were initially associated with a number of clinically overlapping neurodegenerative disorders characterized by the common finding of hypomyelinating leukodystrophy. Over the past 2 years, there has been mounting evidence that *POLR3A*-related disorders are a growing group of clinically and genetically heterogeneous diseases with a wide range of timing at onset (from neonatal period to adulthood) and possible involvement of several neurological and non-neurological systems. The intronic c.1909 + 22G > A variant has been reported in compound heterozygosity in patients with a slowly progressive neurodegenerative disease characterized by late onset and a uniform clinical picture of spastic ataxia (rarely pure ataxia), SCP hyperintensity, and spinal cord atrophy on MRI. Frequent concurrent features (present in at least 20% of cases) were dental problems, kinetic tremor, muscle atrophy, dysarthria, *pes cavus*, ocular problems, thinning of the corpus callosum (TCC), dystonia, and polyneuropathy. Hypogonadism was rare (5%) and leukodystrophy absent [13]. Others [7] have demonstrated that the “common” c.1909 + 22G > A variant is a mutation hotspot.

In this work, we confirmed the strong genotype–phenotype correlation between the c.1909 + 22G4A variant combined with a second, “variable” *POLR3A* mutation and juvenile-adult-onset spastic ataxia with SCP hyperintensity and spinal cord atrophy. We have here detailed 10 new cases that support this association and provided further details on the related clinical, genetic, and neuroimaging features. While the “common” c.1909 + 22G4A variant affects skipping by introducing a cryptic donor splice site and likely impairing mRNA abundance [10], the other variants are straightforwardly pathogenic leading to early protein truncation or resulting in a frameshift with a premature stop codon.

Our cohort confirmed that intentional tremor and dysarthria are frequent associated features, whereas dental anomalies, dystonia, and polyneuropathy are only occasionally detected.

One of our cases (patient 2) presented with pure pyramidal involvement. To date, only one subject with a similar pure HSP phenotype has been reported, and this subject had a different genotype in the “variable” allele [19]. The MRI features of our cohort coincided with those reported in previous series: the key feature was SCP hyperintensity, shown by 70% of our patients, followed by cerebellar atrophy (50%), spinal cord atrophy (50%), and TCC (30%); corticospinal tract hyperintensity was an inconstant feature. Thus, our data further reinforce the message that apparently sporadic HSP patients should be investigated for SCP hyperintensity, before ordering *POLR3A* studies. Previous authors have demonstrated that the c.1909 + 22G > A variant is a relatively common cause of disease in cerebellar ataxia/HSP patients [7]. Our study seems to confirm this in the Italian ataxia/HSP population, too, even though *POLR3A* accounts for less than 2% of the solved cases in our cohort of patients with inherited ataxia and HSP undergoing NGS (FMS and AT personal communication).

Our data expand the genetic heterogeneity of this relatively frequent form of spastic ataxia. Our series includes the first patient found to carry a large deletion involving exons 14–18; this was suspected on the basis of lower coverage in NGS studies and detected by gene-specific qRT-PCR analyses. To date, intragenic deletions have been described only in *POLR3B*, which encodes another catalytic subunit of Pol III, in two patients with severe infantile hypomyelinating leukodystrophy [20], and, more recently, in *POLR3A* in a patient with early-adulthood onset cerebellar ataxia and cognitive impairment and hypomyelinating leukodystrophy [3]. Since both traditional and massive, NGS-derived sequencing techniques can fail to detect intragenic rearrangements; our finding underlines the usefulness of quantitative techniques in selected cases. Interestingly, our patient with the novel deletion involving exons 14–18 had the most “complex” phenotype observed within this relatively small cohort, showing several neurological and non-neurological features (drug-responsive generalized epilepsy, neurosensory deafness, and lipomas) that, not previously reported in *POLR3A*-related disorders, further expand the phenotype.

In addition to this five-exon deletion, the other loss-of-function mutations identified in this cohort add to the allelic heterogeneity of *POLR3A*-related disorders, but did not seem to influence the clinical presentation.

In conclusion, our study strengthens the genotype–phenotype characterization of the c.1909 + 22G > A compound heterozygous variant and, identifying an intragenic multi-exon deletion associated with new clinical features, expands the molecular mechanisms and clinical spectrum associated with late-onset *POLR3A*-related disorders.

## Supplementary Information

Below is the link to the electronic supplementary material.

Supplementary file1 (PPTX 252 KB)
