# Supplementary material for: POLR3A variants in hereditary spastic paraparesis and ataxia: clinical, genetic, and neuroradiological findings in a cohort of Italian patients
Source: Neurol Sci. 2021 Jul 23;43(2):1071–7. doi: 10.1007/s10072-021-05462-1 (PMC8789690; doi:10.1007/s10072-021-05462-1)
Supplement: Supplementary file 1 — Supplementary file1 (PPTX 252 KB) [file 10072_2021_5462_MOESM1_ESM.pptx]

## Slide 1
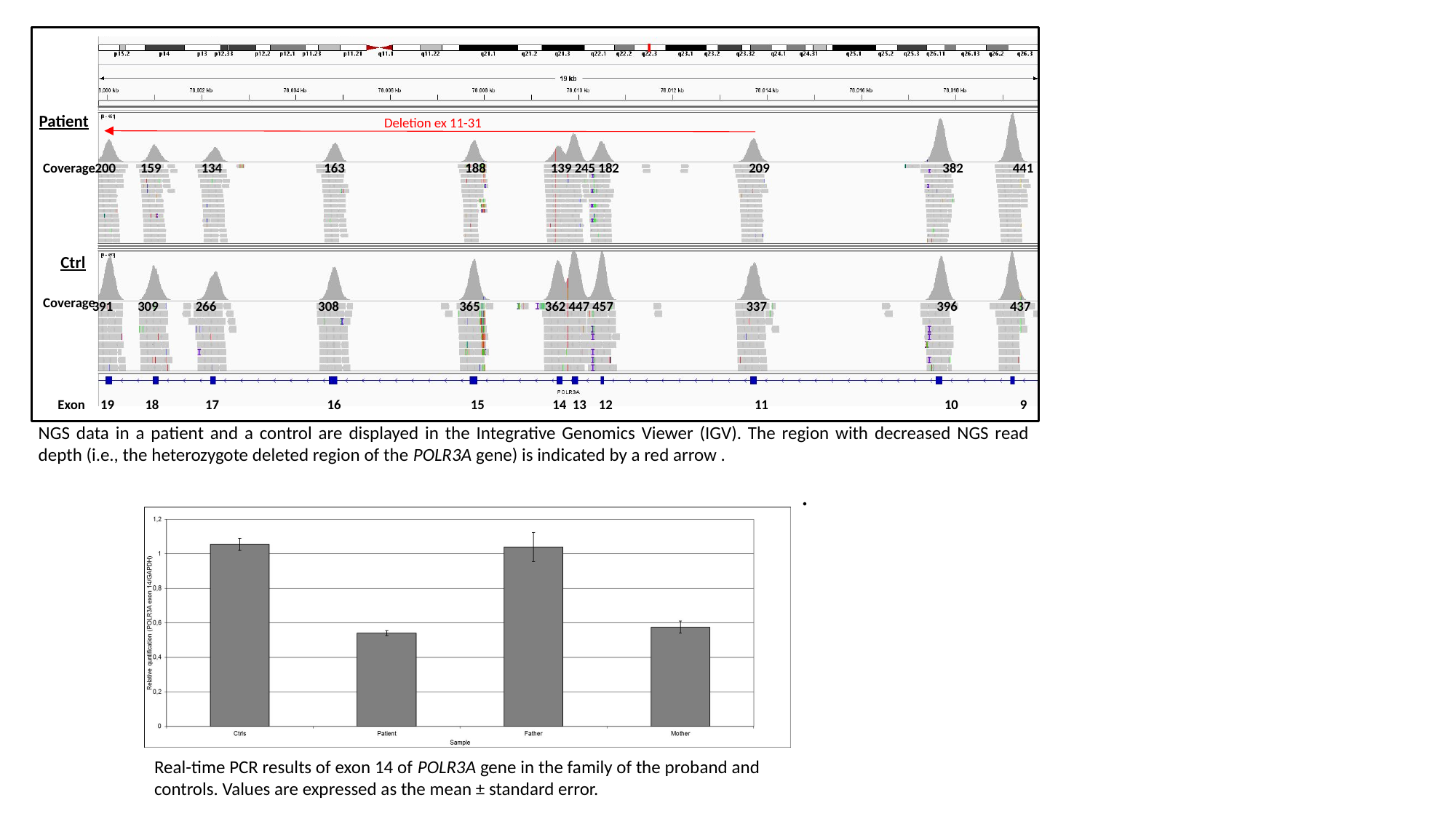

Patient
Deletion ex 11-31
Coverage
200 159 134 163 188 139 245 182 209 382 441
Ctrl
Coverage
391 309 266 308 365 362 447 457 337 396 437
 Exon 19 18 17 16 15 14 13 12 11 10 9
NGS data in a patient and a control are displayed in the Integrative Genomics Viewer (IGV). The region with decreased NGS read depth (i.e., the heterozygote deleted region of the POLR3A gene) is indicated by a red arrow .
.
Real-time PCR results of exon 14 of POLR3A gene in the family of the proband and controls. Values are expressed as the mean ± standard error.
